# Integrated Graphene Heterostructures in Optical Sensing

**DOI:** 10.3390/mi14051060

**Published:** 2023-05-17

**Authors:** Phuong V. Pham, The-Hung Mai, Huy-Binh Do, Vinoth Kumar Ponnusamy, Feng-Chuan Chuang

**Affiliations:** 1 Department of Physics, National Sun Yat-sen University, Kaohsiung 80424, Taiwan; 2 Faculty of Applied Science, Ho Chi Minh City University of Technology and Education, Ho Chi Minh City 700000, Vietnam; 3 Department of Medicinal and Applied Chemistry and Research Center for Precision Environmental Medicine, Kaohsiung Medical University (KMU), Kaohsiung 807, Taiwan; 4 Department of Medical Research, Kaohsiung Medical University Hospital (KMUH), Kaohsiung 807, Taiwan; 5 Department of Chemistry, National Sun Yat-sen University, Kaohsiung 80424, Taiwan; 6 Physics Division, National Center for Theoretical Sciences, Taipei 10617, Taiwan; 7 Center for Theoretical and Computational Physics, National Sun Yat-sen University, Kaohsiung 80424, Taiwan

**Keywords:** integrated graphene heterostructure, optical sensing, plasmonic, optical waveguide, spectrometer, synaptic system, perspective

## Abstract

Graphene—an outstanding low-dimensional material—exhibited many physics behaviors that are unknown over the past two decades, e.g., exceptional matter–light interaction, large light absorption band, and high charge carrier mobility, which can be adjusted on arbitrary surfaces. The deposition approaches of graphene on silicon to form the heterostructure Schottky junctions was studied, unveiling new roadmaps to detect the light at wider-ranged absorption spectrums, e.g., far-infrared via excited photoemission. In addition, heterojunction-assisted optical sensing systems enable the active carriers’ lifetime and, thereby, accelerate the separation speed and transport, and then they pave new strategies to tune high-performance optoelectronics. In this mini-review, an overview is considered concerning recent advancements in graphene heterostructure devices and their optical sensing ability in multiple applications (ultrafast optical sensing system, plasmonic system, optical waveguide system, optical spectrometer, or optical synaptic system) is discussed, in which the prominent studies for the improvement of performance and stability, based on the integrated graphene heterostructures, have been reported and are also addressed again. Moreover, the pros and cons of graphene heterostructures are revealed along with the syntheses and nanofabrication sequences in optoelectronics. Thereby, this gives a variety of promising solutions beyond the ones presently used. Eventually, the development roadmap of futuristic modern optoelectronic systems is predicted.

## 1. Introduction

Converting light into electrical signals is technically performed by using optoelectronic systems—vital components for capturing and envisaging information in optics. Optical sensing systems typically detect photons, which hold a key role in actual applications, e.g., industry, agriculture, military, or health, [1,2,3,4,5,6,7,8]. Considerable studies have grown around the theme of several semiconductors, e.g., Ge, GaAs, or SiGe applied for optical sensing systems [9,10]. However, they showed unexpected efficiency with low light transparency and flexibility, narrow bandgap, etc. Thus, this resulted in deterioration in the performance of light absorptivity and responsivity, thereby inducing low quantum efficiency, as well as low detection ability. These drawbacks significantly affect integrated detection devices, particularly future optical sensing systems.

**Figure 1:**
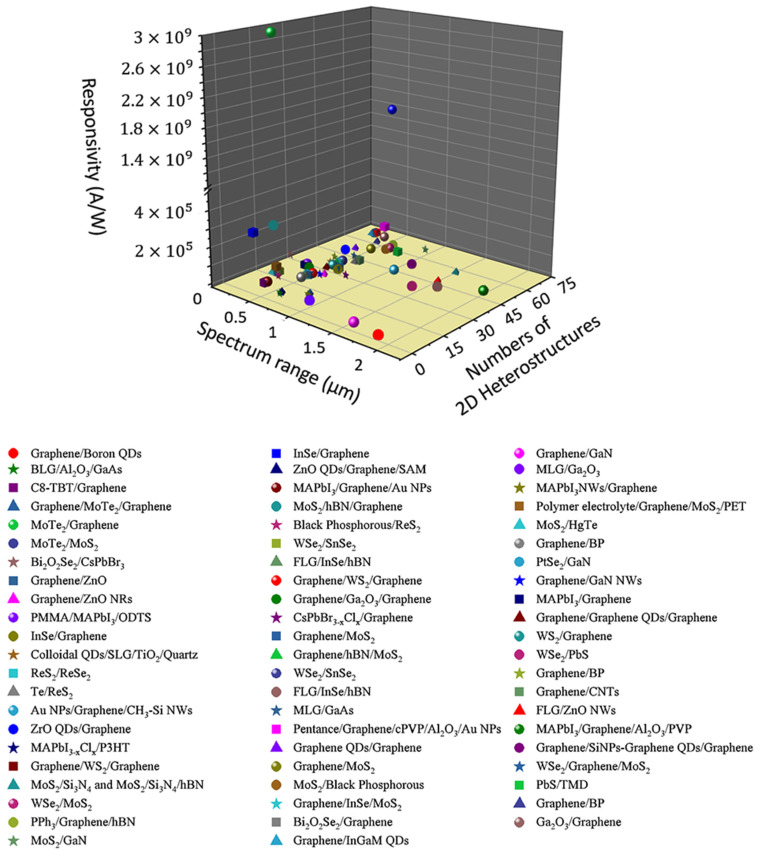
Merits of the optical responsivity performances of optical sensing systems under the broad absorption band (UV to IR) using representative integrated 2D heterostructures. Adopted with permission from Ref. [18]. Copyright 2022 American Chemical Society.

Beyond the limited semiconductors above, silicon (Si) has shown to be an exceptional candidate with efficient properties, low dielectric constant, and indirect bandgap, aiming for fast operation and good responsivity [11]. Besides that, using low-dimensional materials, including graphene from one-dimensional (1D) (carbon nanotube, graphene nanoribbons (GNRs)) to two-dimensional (2D) graphene, have also been demonstrated to bring improvement to the performance of optoelectronics. 2D graphene was discovered by Novoselov et al. in 2004. Since then, all manner of strange and exotic phenomena has been observed in graphene. These include: the Kelin effect, in which charge carriers are able to pass right through high potential barriers, as if they were not there; also shown was the survival of the half-integer quantum Hall effect at room temperature. Moreover, the combination of high conductivity and inherent flexibility make 2D graphene attractive for optoelectronics [12,13,14]. Unlike 2D graphene, 1D graphene shows novel physics behaviors that depend on boundary configuration, typically similar to GNRs, which were introduced as theoretical models by Nakada et al. and Wakabayashi et al. [15,16]. For example, the configuration of zigzag GNRs exhibits special electrical properties, as well as semi-metal properties; armchair GNRs are semi-conducting in nature, wherein the band gap is inversely proportional to their width. This leads to their special electronic structures, as well as their characteristics of high electrical conductivity, high thermal conductivity, and low noise. Therefore, the use of low-dimensional graphene in devices is extremely promising [17]. Thereby, combining Si with low-dimensional material, e.g., graphene can be potentially useful in electronics and optoelectronics with high compatibility in complementary metal-oxide-semiconductor (CMOS). Here, Si-based representative optical sensing systems and related industrial applications (plasmonic devices, optical waveguides, optical spectrometers, and optical synaptic systems) are highlighted. Heterostructures used for optical sensing, based on graphene and other materials, are also summarized (Figure 1). Eventually, some perspectives ahead shed new light on these developments.

## 2. Optical Sensing Mechanism of Integrated Graphene Heterostructures

To date, new optical sensing systems have been revealed, varying in their structures, morphologies, materials used, and physical properties, which are dependent on the photoelectric effect of sensing system. Recently, a heterostructure-based optical sensing system using a multilayer fluorinated graphene structure treated by SF_6_ gas precursor, whose detectivity varied from ultraviolet (UV) to mid-infrared range (0.25–4.3 µm) has been reported and resulted in good detectivity and responsivity at an acceptable on/off ratio in industry [19]. This could be a good strategy for innovative explorations of future integrated graphene heterostructure optical sensors. In another study, Wan et al. utilized an Al_2_O_3_ passivation layer-covered graphene layer to form a new junction with thin arrays in order to enhance UV absorption [20]. As a result, it obtained a good responsivity value (0.14 A/W) at 0.2–0.4 μm, with a fast response time (5 ns), high detectivity, at $1.6\times 10^{13}$ Jones, and a high on/off ratio ($1.2\times 10^{6}$) at 0.365 μm wavelength with the internal quantum efficiency (>100%). Moreover, a nanostructured device was suggested, which was based on an Au-decorated graphene–hBN–graphene heterostructure [21]. By using this new heterojunction, it formed plasmon-induced hot electron carriers and enhanced the hot electrons through the absorption of light.

**Figure 2:**
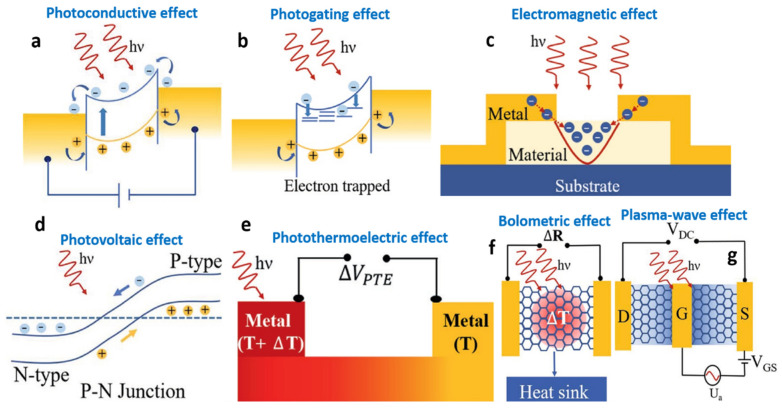
The general mechanism of low-dimensional material-based optical sensing (**a**) Photoconductive effect, (**b**) Photogating effect, (**c**) Electromagnetic effect, (**d**) Photovoltaic effect, (**e**) Photothermoelectric effect, (**f**) Bolometric effect, and (**g**) Plasma-wave effect. (**a**–**g**) are reproduced with permission from Ref. [2]. Copyright 2021 Wiley.

**Figure 3:**
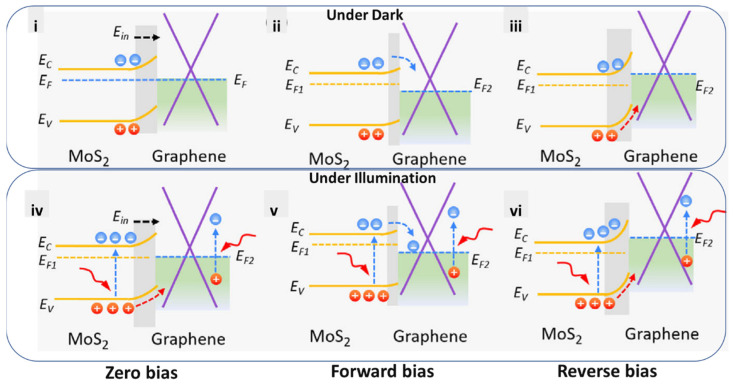
The optical sensing mechanism depicted for representative graphene-integrated heterostructures (MoS_2_/graphene/Si). (**i**–**iii**) The band diagrams under zero bias, (**i**) forward bias, (**ii**) reverse bias, and (**iii**) in the dark. (**iv**–**vi**) The band diagrams under zero bias, (**iv**) forward bias, (**v**) reverse bias, and (**vi**) under illumination. Reproduced with permission from Ref. [3]. Copyright 2021 Wiley.

In the same issue, Liu et al. revealed a new junction, thereby contributing to the improvement of the response time, lowering dark current, and adjusting hot electrons in optoelectronics [15]. In another work, a research group revealed the possible optical effects of combined heterojunctions, e.g., photovoltaics, photogatings, photoconductives, photo-thermoelectrics, plasma waves, or bolometrics (Figure 2 and Figure 3) [1,2,3]. Additionally, Massicotte et al. explained the ultrafast optical sensing of conventional junctions [22]. Besides photodetections, the pros and cons of heterojunctions have also been discussed [3]. By monitoring the design methods of combined optical sensing systems, they could overcome the existing limitations by employing new nanostructures.

Concerning the exploration of plasmon-induced hot electrons in heterostructure junctions, the outcomes of the heterostructure devices need to be figure out in broad applications composed of spectroscopies, energy harvests, optic communications, medicals, or synaptic systems, in which combined nanostructures provide a platform to employ quantum effects. For instance, the ultrafast cooling of hot carriers can be tuned in the architecture of the junction thereby enhancing quantum efficiency. Hence, this mini-review will discuss graphene-based heterojunctions for optical sensing systems and their applications, based on various factors, such as plasmonic systems, photonic systems, and hot electron effects.

## 3. Representative Application of Integrated Graphene Heterostructures

**Figure 4:**
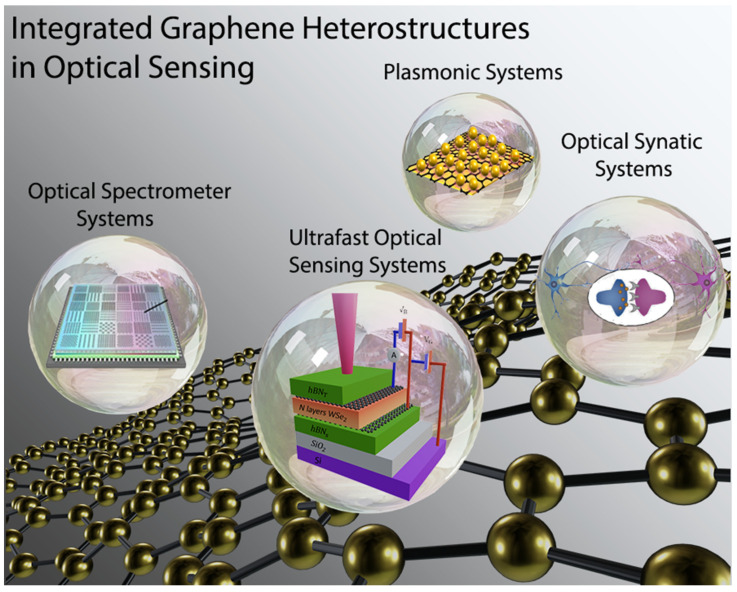
The foremost representative applications of integrated graphene heterostructures in optoelectronics.

The application associated with 2D materials, such as graphene and others (e.g., transition metal dichalcogenides (TMDs), silicene, 2D metal carbides and nitrides) are significantly reliant on how these materials integrate with others. During the last century, the diversity of optoelectronics, systems from one dimension (e.g., fullerene, carbon nanotube, or metal nanowire) to two dimensions (e.g., graphene, TMDs, or hBN), was explored. In this section, we address the foremost representative applications, where integrated graphene heterostructures constitute a primary mission (Figure 4).

### 3.1. Ultrafast Optical Sensing Systems

**Figure 5:**
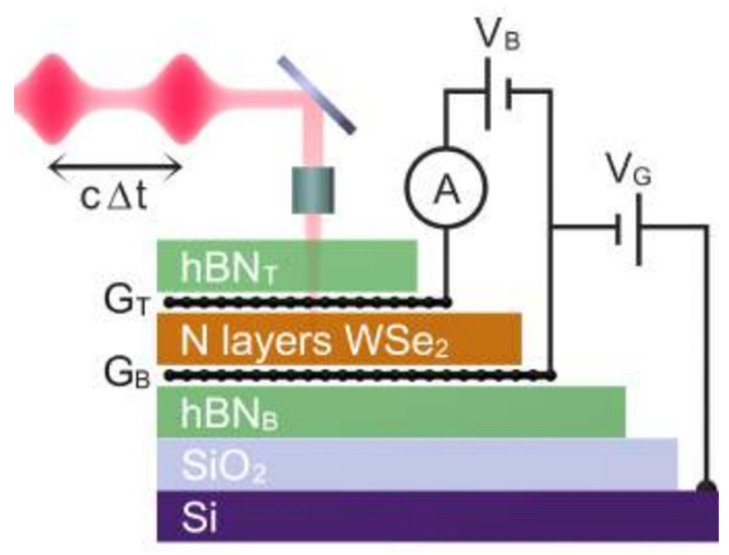
hBN/graphene/WSe_2_/hBN-based integrated heterostructures for ultrafast optical sensing. Reproduced with permission from Ref. [22]. Copyright 2021 Nature Publishing Group.

Ultrafast optical sensing in the range of picoseconds is an extraordinary evolution in next-generation optical sensing systems. Massicotte et al. recently reported this extraordinary performance of an integrated optical sensing system by combining it with hBN–graphene–${\mathrm{WSe}}_{2}$–graphene–hBN, and the result was an ultrafast optical sensing response up to the picosecond range, which has been reported on and presented on by Massicotte et al. (Figure 5) [22]. They, furthermore, investigated the quantum efficiency of more than 70% at the illuminated wavelength of 0.759 µm, with a rise time of 5.5 picosecond (ps), and NEP was reported to be $5\times 10^{-12}\;W/{\mathrm{Hz}}^{1/2}$. These findings lead us to reveal new ways of designing optoelectronics devices for potential applications in optical communications. In conventional optical sensing systems, high photon absorption and photoconductance were already reported, but this ultrafast optical sensing response was not observed before. Therefore, Massicotte et al.’s discovery is considered a milestone in the optoelectronic device field, where they used a sandwiched structure, along with different bandgap materials, to gain ultrafast charge extraction at the device interfaces. The photoresponse process was regarded as exciton recombination in this study [22]. In the conventional graphene/TMD interfaces study, exciton dissociation time was reported as 0.7 ps in the bulk structure of TMDs. Whereas, for a 2D nanostructure material-based interfacial transfer, it was reported to be approximately 1 ps.

### 3.2. Plasmonic Systems

**Figure 6:**
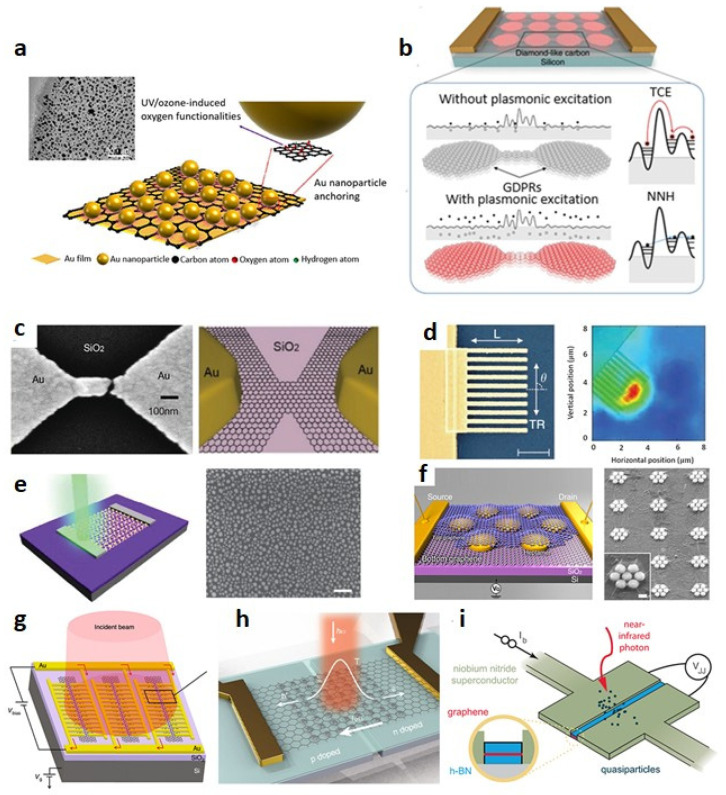
Graphene-based plasmonic systems. (**a**) Au NPs/rGO/Au thin films-integrated plasmonic optical systems. Reproduced with permission from Ref. [25]. Copyright 2021 Springer. Graphene-integrated hybrid structures for optical sensing. (**b**) Graphene–disk plasmonic resonators connected by quasi-1D graphene nanoribbons. (**c**) An Au nanogap antenna with graphene in the gap. The scale bar is 100 nm (5 μm for inset). (**d**) Finger type Ti/Au plasmonic nanostructures on graphene. The right figure indicates the photovoltage map, illuminated with 514 nm light with transverse polarization. The scale bar is 1 μm. (**e**) Graphene photodetector with AuNPs (**left**) and SEM image of AuNPs on a graphene surface (**right**). The scale bar is 100 nm. (**f**) A single Au heptamer sandwiched between two monolayer graphene sheets (**left**) and a SEM image of a Au heptamer (**right**). The scale bar in the inset of the right figure is 100 nm. (**g**) Au-patched graphene nano-stripes for utilizing maximum metal–graphene interfaces for enhanced photocurrent. (**h**) A graphene photodetector integrating both optical heating enhancement (via gap plasmonic structures) and electrical junction enhancement (via split gates). (**i**) A single photon detection device using a Josephson junction. (**b**–**i**) Reproduced with permission from Ref. [24]. Copyright 2022 Springer.

Plasmonic excitation is an effective way to produce hot electrons in metals [23,24,25,26], through the light absorption at low energy levels (Figure 6) [27,28]. These plasmon excitations relax in two ways: through radiation of emitting photons and through non-radiation processes, forming electron–hole pairs as a result of plasmon resonance [29]. These plasmonically generated hot electrons are then collected by a Schottky metal–semiconductor junction, identical to the photothermionic emission electron collector. Hence, the integration of plasmonic devices by 2D semiconductors can be used to boost the optical absorption and photoresponsivity of the device, such as graphene integrated plasmonic devices, which were reported to have a high quantum efficiency, being 1500%, along with enhanced photoresponse of graphene at varying resonance frequencies [30]. However, in metallic nanostructures, the plasmonic resonance is only in the visible range of wavelengths. Moreover, 2D materials themselves can have the potential for promising optical sensing efficiencies by using their intrinsic plasma mode instead of integrating them with other materials [31]. However, this intrinsic plasma mode of 2D materials is somehow different from metals, since these 2D materials can tune their bandgaps and properties by doping, which would affect the optical sensing range up to the terahertz radiation limit. It can also cause surface plasmons to be confined to a volume smaller than their diffraction limit, resulting in strong light–matter interactions [32]. This strategy serves another advantage in that the surface plasmons of 2D materials can enhance their lifetime by hundreds of optical reputations, thus overcoming the metal-based plasmonic devices’ limitations and providing wider opportunities for quantum devices [33]. Moreover, polaritons are generated by the coupling of light and other elementary particles, such as phonons, plasmons, or excitons, which carry electric dipoles in a system. Hence, by utilizing these intrinsic polaritons’ resonances, light–matter interaction can also be enhanced [34]. Localized plasmon modes are generated by the confinement of light in a smaller volume than its diffraction limit. Hence, by using this concept, patterning graphene in micro-ribbons also generates localized plasmon resonance in the terahertz region, which is not possible in conventional light–graphene interaction [33].

Moreover, the external voltage biasing can be used to tune the plasmon resonance peak level and amplitude to obtain the required detection wavelength range. Monolayer graphene has the drawback of carrier concentration, which leads to weak amplitude and, hence, low plasmon resonance. The multilayer graphene offers a crystal-like structure, with its layers separated by insulating material, which overcomes the carrier concentration problem and, hence, enhances the plasmon resonance effect [35]. This response is correlated with the Dirac fermion law of plasmonic resonance, which is quite different from plasmonic resonance in semiconductors [35]. As a result, the concept of graphene-based plasmonic resonance and the hot electron effect could be applied to the advanced next generation of high-performance optical sensing systems, with detection ranges ranging from UV to terahertz.

### 3.3. Optical Waveguides and Cavities Systems

**Figure 7:**
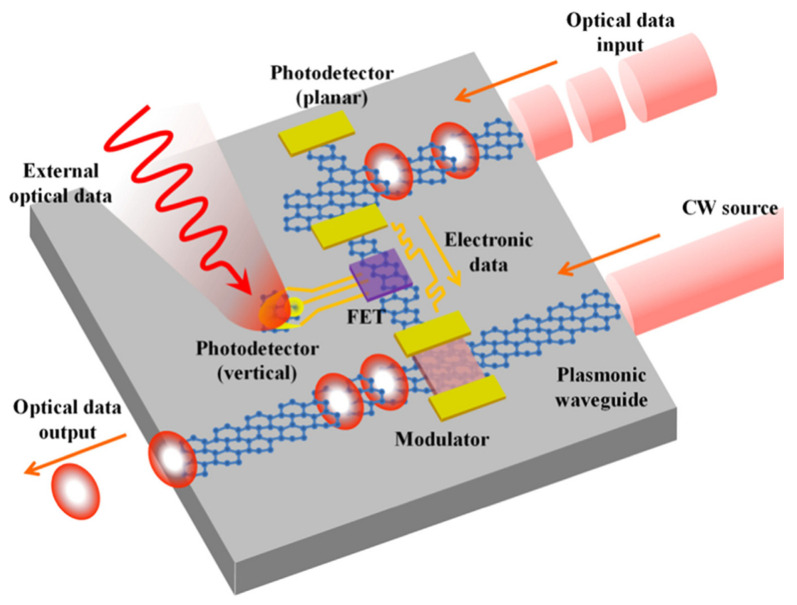
An optical waveguide using graphene heterostructures for electronic photonic integrated circuits configured on a chip. Reproduced with permission from Ref. [39]. Copyright 2018 Elsevier.

A waveguide is a device to guide wave propagation with a limited loss of wave energy in a particular direction. The wide wavelength ranges from near IR to far IR demand for Si photonics to be utilized in various applications in optical communication (Figure 7) [36,37,38,39]. Guo et al. and Yin et al. have also investigated waveguide-based hybrid nanostructure optical sensing systems, having combinations of black phosphorous (BP)–${\mathrm{Al}}_{2}O_{3}$ and Si, with graphene–${\mathrm{Al}}_{2}O_{3}$_,_ to be used as physical links in devices to enhance their responsivity to 0.3 A/W and 0.4 A/W at the illuminated wavelength of 2 µm and 1.55 µm, respectively [38,40]. In comparison with conventional semiconductor materials, 2D materials have a much higher optical coefficient. However, this situation may be handled by improving the interaction between light and 2D material and adjusting the absorption loss by integrating the device with optical waveguide photonic structures [37,41]. In another study, graphene was reported to be integrated with Si optical waveguide, which resulted in a maximum photoresponsivity of 0.15 A/W, with 20 GHz bandwidth at the operating wavelength range of 1.3 µm to 1.6 µm [37]. However, for effective optical absorption in graphene, much longer waveguides are required, but this will enhance the capacitance of metallic contacts and limit the bandwidth to a few GHz. On the other hand, microcavity-integrated devices are efficient, but at the cost of high absorption bandwidth.

### 3.4. Photonic Crystal (PhC) Sensors Based on PhC Nanocavities and Waveguides

The concept of PhC was first introduced by E. Yablonovitch and S. John in 1987, and it possesses a periodic dielectric structure, and the capability manipulates the flow in a controlled way [42,43,44]. Recently, many PhC-based devices have been widely used in optical applications, such as electro–optical modulators, switches, or delay devices. Therein, PhC sensors seem to be much more popular, owing to their outstanding characteristics, such as high measurement sensitivity, flexibility in structural design, and CMOS compatibility [44,45].

As a representative optical structure, a PhC nanocavity is formed by introducing point defects in the orderly arranged lattices. It exhibited strong light confinement and a long photon lifetime, which is represented by the value of the quality factor (Q-factor) [46,47]. A high Q-factor means great enhancement of the interaction strength between the optical field and the material of the defect region. As for sensing applications, the enhanced interaction effect gives an increase to an optical mode of the PhC nanocavity, with a resonant wavelength that is highly sensitive to the local variations in its surrounding medium, and this makes the PhC nanocavity a promising high-sensitivity optical sensor [44,48]. Recent studies by Takada et al. and Noda et al. purposed the improvement of a design with a hexapole Si PhC nanocavity. Concerning this, the system is based on a Si slab with thickness t = 250 nm, and Si PhC is a triangular lattice of circular air holes of radius $R_{o}$ ≈ 133.1 nm with lattice constant a = 434 nm, in which the lack of a single hole leads to a point defect and forms an hexapole PhC nanocavity. In addition, the six holes near the point defect have a smaller radius, $R_{1}\approx 106.8\;\mathrm{nm}$. This structure obtained a Q-factor of $1.2\times 10^{6}$ [49]. In another study, the defect of the Si PhC nanocavity consisted of three losing air holes, called the L3 Si PhC nanocavity, which is constituted of a = 410 nm, a hole radius (R = 108 nm), and a thickness (t = 220 nm). The Q-factor enhancement of this structure was observed at $5\times 10^{6}$ after parameter optimization [50]. Meanwhile, Kassa-Baghdouche et al. composed a slotted Si PhC waveguide consisting of a 2D hexagonal PhC lattice of air holes etched in a Si membrane. The slotted PhC waveguides form by omitting the central row of the air holes and replacing it with a slot of width 0.33a (a = 1060 nm), and the radius of the air hole is 0.29a. The author has indicated and demonstrated a high sensitivity of more than 1150 nm per refractive index unit, with an insertion loss level of −0.3 dB [51]. The attained results and structures above have proven the strength of the PhC nanocavity and waveguide, and these structures could improve optical applications.

**Figure 8:**
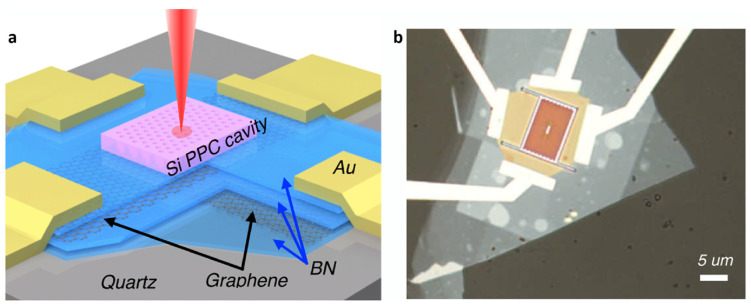
Schematic (**a**) and OM image (**b**) of Si PPC/BN/graphene heterostructure-based photonic crystal nanocavity. (**a**,**b**) are reproduced with permission from Ref. [54]. Copyright 2015 American Chemical Society.

Nevertheless, the integration of the dielectric PhC nanocavities or waveguides with the high Q-factor increases modulation strength in optical resonators, but these devices suffer from intrinsic narrow bandwidth and weak electro–optical properties [52,53]. Therefore, a new modulator architecture, based on the graphene heterostructures integrated with a planar PhC cavity to overcome, as well as further, enhancement of sensitivity in the optical systems, has been considered. Yuanda et al. have also investigated a Si PPC/BN/graphene heterostructure-based photonic crystal nanocavity, utilizing a liner with three missing air holes in the Si PhC nanocavity (Figure 8). As a result, a modulation depth of 3.2 dB is observed, with a cut-off frequency of 1.2 GHz, and the cavity bandwidth exceeds 600 GHz. This proved that the strong electro–adsorptive effect of graphene in the PhC nanocavity could enable high performance, electro–optic modulators with low power consumption, and high speed, which would be very expected in optical systems [54]. Moreover, many other graphene heterostructures have shown outstanding properties, and, when totally investigating the optical systems, such as the graphene–tungsten oxyselenide heterostructure, we obtained a high carrier mobility of $2000{\;\mathrm{cm}}^{2}{\;V}^{-1}s^{-1}$ and a hole density $3\times 10^{13}{\;\mathrm{cm}}^{-2}\;$ at room temperature [55]. Besides, many TMDs and graphene have also indicated that they have an optical adsorption coefficient higher than conventional Si and GaAs [18]. Therefore, integrated heterostructures in the optical application have extreme potential, not only to overcome some drawbacks of devices based on PhC nanocavity and waveguides, but, also, they can bring better performance.

Thereby, a potential optical research branch by the integration of graphene heterostructures into the aforementioned optical system (PhC nanocavity and waveguide) could shed light on the unexplored one in achieving superior features in new physics and optical sensing.

### 3.5. Optical Spectrometer Systems

**Figure 9:**
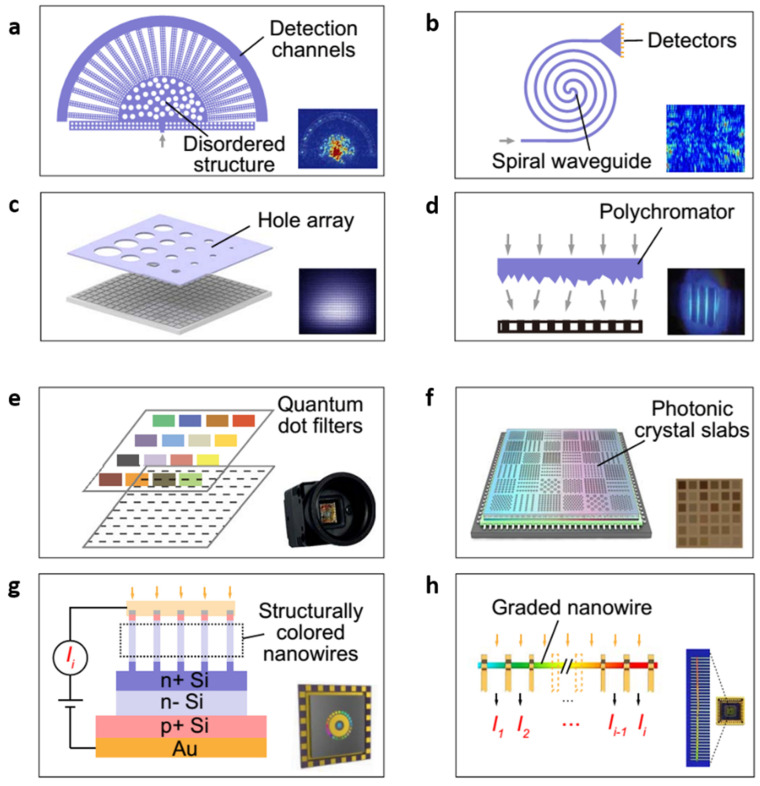
Computational optical spectrometers adopted for spectral-to-spatial mapping: (**a**) a disordered photonic chip [57], (**b**) a spiral waveguide [58], (**c**) a dispersive hole array [59], (**d**) a polychromator [60], (**e**) colloidal quantum dot mixtures [61], (**f**) photonic crystal slabs [62], (**g**) arrays of structurally colored nanowires [63], (**h**) a single compositionally engineered nanowire [64]. Reproduced with permission from Ref. [65]. Copyright 2021 AAAS.

An optical spectrometer is used to identify the spectral parameters in a physical mechanism (Figure 9) [56,57,58,59,60,61,62,63,64,65]. To integrate it with nanostructures, a spectrometer is required to minimize its physical dimensions, such as size and weight [66,67,68]. These compact devices can be used for commercial applications, such as security and health. These spectrometers can also be developed on a 2D material system to replace the existing electronic systems with integrated nanostructured devices. Yuan et al. reported a high photoresponse mid IR spectrometer by tunable phosphorous optical sensing systems in the range of 2 to 9 µm, with a footprint area of 9 to 16 μm^2^ [69]. Without a transparent top gate of graphene and the strong encapsulation of hBN, along with the appropriate density of BP, the device’s exceptional efficiency would indeed be impossible. The BP PDs’ responsivity depends on both the illuminated light wavelength and the displacement field subjected to the external biasing, denoted as R (λ, D). To rebuild the spectrum of the incident light, these data are compared to a pre-calibrated matrix of responsivities for standard solutions. A chip-based integrated spectrometer can also be created using an array of PDs and advanced algorithms and computational techniques. Along with establishing a novel technique for miniature spectrometry, an equal effort should be made to modify the individual components of existing spectrometers. Shrestha et al. investigated an interferometer based on graphene to measure the mid-IR range incident light spectrum by the phenomenon of tunable transmission [70]. The graphene surface of this device is made up of metallic nanostructures and dielectric cavities, whose reflection spectrum is adjustable by applied gate voltage. Generally, the development of the spectrometer by utilizing 2D nanofilms could pave more paths in the advancement of optoelectronic devices.

### 3.6. Optical Synaptic Systems

**Figure 10:**
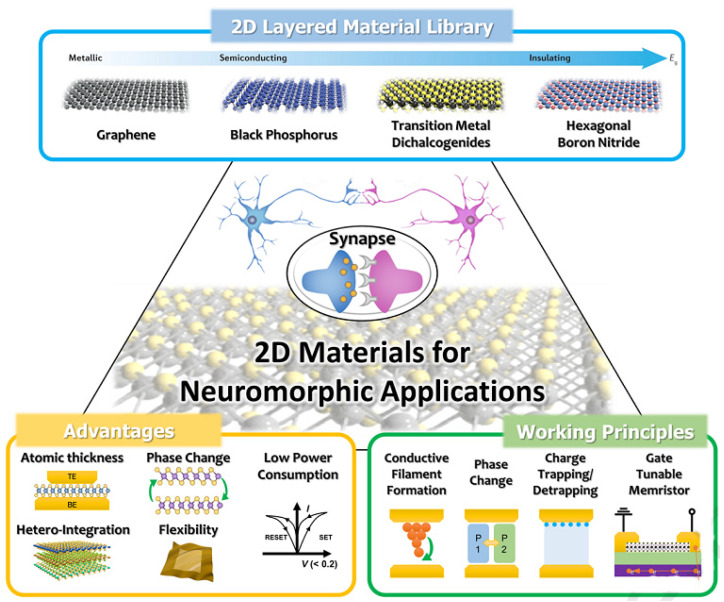
Optoelectronic synaptic systems. An overview of neuromorphic applications, based on representative 2D materials and graphene/MoS_2_ nanostructure-based synaptic devices. Reproduced with permission from Ref. [90]. Copyright 2020 Elsevier.

The cognitive action of a human system depends on neurons’ interactions, called synapses, presented in Figure 10, on behalf of which the artificial memory circuits, having a neuromorphic computing system, are called synaptic devices [71,72,73,74,75,76,77,78,79,80,81,82,83,84,85,86,87]. In these kinds of devices, the strength of the neuronal interactions is comparable to the electrical response of the device, such as the change in resistance by employing external voltage or light illumination, called synaptic weight. The ORRAM devices include both neuromorphic and optical computing, which can be controlled by both volatile and non-volatile methods through the adjustment of the device switching mechanism. Light-dependent synaptic characteristics, such as spike rating-dependent plasticity, long-term plasticity, spike timing-dependent plasticity, and short-term plasticity, can be represented by these synaptic systems [75,76,79]. 2D materials are considered to be potential candidates for the growth of volatile and non-volatile ORRAM synaptic devices due to their strong light–matter interactions, which result in efficient device performance, such as high responsivity, wide photoresponse range, high on/off ratio, enhanced energy efficiency, and fast switching speed. The first representation of these devices was reported by nanostructures of carbon and graphene with their light-dependent synaptic behavior [76]. The gate voltage and the optical pulse govern the channel conductance in this carbon and graphene-based device. The biological synaptic weight is used to depict the various conductive modes. WSe_2_/Boron-doped Si-nanocrystals were employed as an ORRAM synaptic system, with optical absorption in the range of UV-near infrared (NIR) and a power consumption of around 75 fJ [77]. This furthered the means for the development of low-power artificial visual intelligence that mimics the human eye. Moreover, the image sensors’ capabilities are broadened to include a variety of potential applications. In 2020, Mennel et al. investigated integrated image sensors as tunable matrices for an artificial neural network (ANN) [78], with high detection speed and fast processing of optical images, which can be optically written on a chip with a capacity of 20 million bits/s and is simply adaptable across various ultrafast machine applications. Beyond Moore’s law, circuit design, based on CMOS, plays a critical part in the technological revolution [88]. Goossens et al. demonstrated a high performance, wide band range, and sensitive image sensor and operated a digital camera in a range of UV to infrared using a monolithic CMOS-integrated circuit aided by CVD graphene on Si [89]. As a result, it could be used in different disciplines, including integrated photonics, high-frequency computing, and sensory matrices. The replication of neural signal transmission in humans and animals by constructing comparable synthetic FEO is a very exciting study issue for further understanding the activity principles of the human body, such as those involved in the neural regions, muscular regions, the brain, etc. From there, creating a multi-functionally artificial neural system that integrates multimodal plasticity, memory, and learning-based functionalities to simulate neuromorphic computation is the next step. Yu et al. (Figure 10) introduced a biomimetic mechano–photonic artificial synapse network with synergistic physical and optical plasticity in 2021 [82]. An optical transistor, based on graphene/MoS_2_ nanostructure and an integrated TENG, were introduced in this optical synaptic system. Optoelectronic synaptic features, such as post-synaptic photocurrent, photoconductivity, and light sensitivity, were well modulated by controlling charge transfer in graphene/MoS_2_ nanostructures with triboelectric potential. The mechanical energy and illuminated light pulse effects show spatial and temporal information to illustrate photonic synaptic plasticity. The artificial neural network also demonstrated good image recognition accuracy of approximately 92%, owing to the mechanical plasticization. This engineered synapse holds a lot of promise for implementing various interactions, imitating the nervous system, and advancing artificial intelligence.

## 4. Conclusions and Perspectives

**Figure 11:**
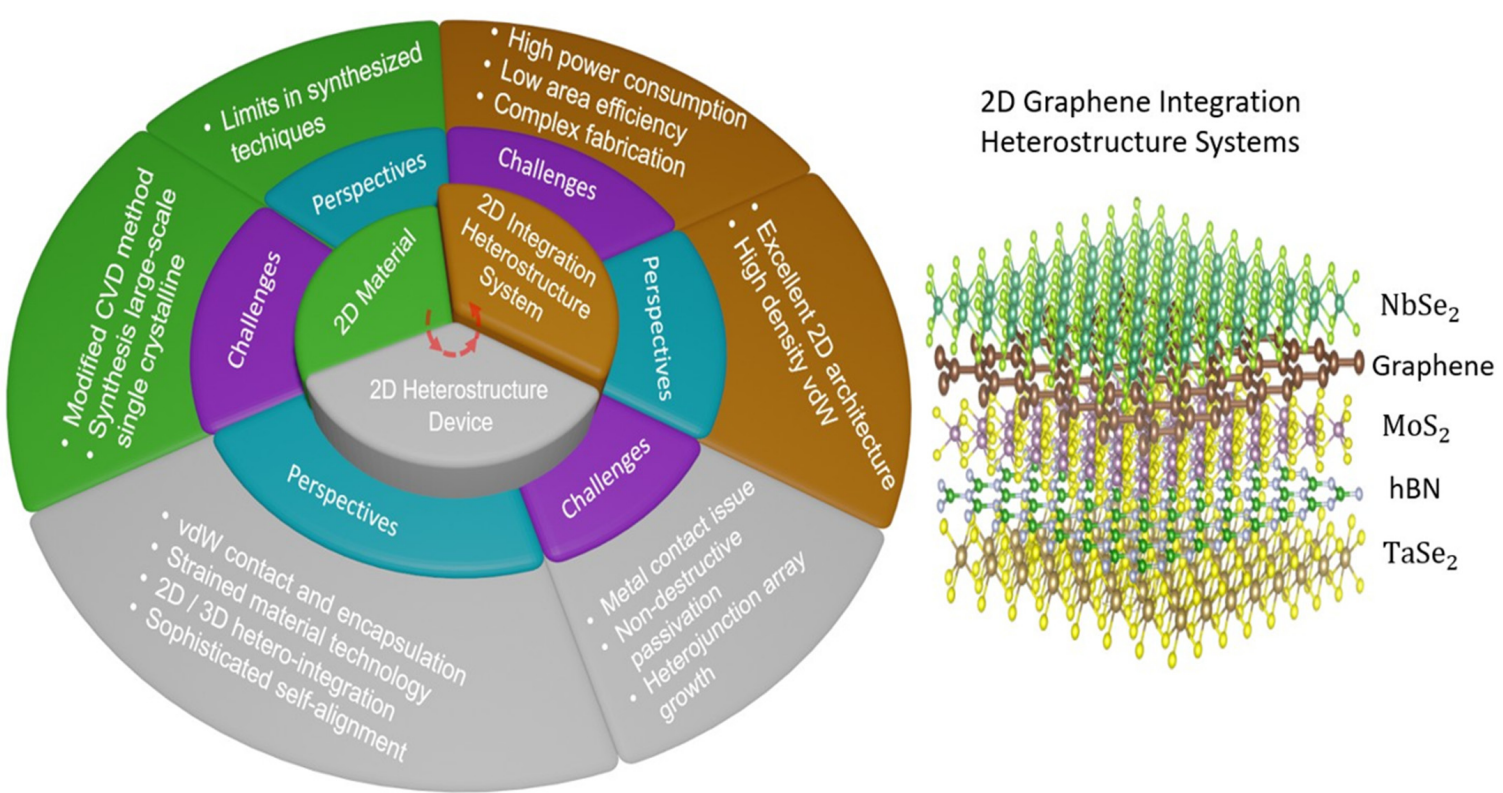
A possible roadmap of 2D graphene integration heterostructure systems-based future optoelectronics: perspectives for the development and challenges from the material synthesis step to the integrated system step.

**Figure 12:**
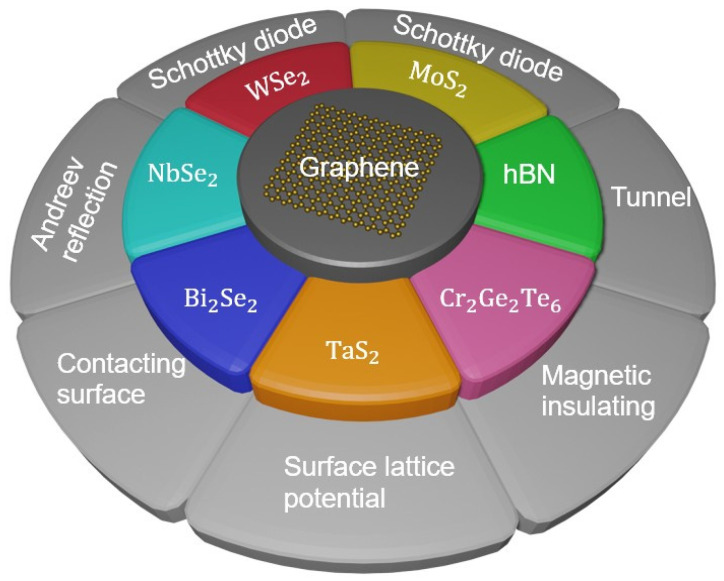
The promising research branches of new graphene/2D materials-integrated heterostructure stacks.

The most recent breakthroughs in integrated graphene heterostructures-based optical sensing systems, as well as their real applications in manufacturing and everyday life, were briefly described. However, realizing the exceedingly increased performance of optical sensing systems on a wafer scale remains difficult. Aside from that, the combination of 2D materials and Si microelectronics could provide a potential technological approach to efficient and cost-effective optical sensing systems, which could be a significant component in next-generation electronics and optoelectronics. In addition, a prediction roadmap and perspectives and challenges of integrated 2D heterostructure-based future electronics and optoelectronics have been revealed by Pham et al. (Figure 11 and Figure 12) [18].

Determining layering dynamics is another difficulty that could lead to novel ways for tuning optical sensing and the spin filtering effects in 2D staked structures. The objective of the heterostructure approach is to produce substantial synthesis and integration processes that are comparable to current Si technology. Despite many outstanding breakthroughs in many fields of optoelectronics that are constrained to narrow circuit-based single devices, inherent challenges, such as material synthesis, device fabrication, and CMOS integration, remain.

One of the most important aspects in determining the performance of optical sensing is called photoresponsivity, which is in the range of UV to far IR. Major developments have recently been made in the exploration of new efficient 2D materials. The most efficient 2D heterostructures used for optical sensing are graphene, BP, low energy band gap TMDs, tellurium, and hybrid 2D heterostructures. Furthermore, it has been reported that the photoresponsivity and yield of these materials have been significantly improved [1,2,3,91,92]. In addition, some parameters that are critical to practical applications of optical sensing, e.g., NEP and wide spectral range photodetectivity, still need more focus.

Integrated graphene heterostructures for optoelectronic device applications have come a long way in recent years. In this chapter, we briefly discussed the fundamentals and potential applications of this framework. In optoelectronics, hybrid materials, such as integrated graphene heterostructures, play a key role in the creation and transmission of photocarriers. The existing state outputs for advanced optoelectronics employing heterostructures are essentially established. However, there are still problems to be addressed from laboratory experiments to actual industrial applications. Beyond the standard, we focus on some related hot topics for future developments and problems, with the goal of providing direction in this rapidly evolving industry.

To begin with, the synthesis of integrated optical sensing systems is the primary concern in the production process of empowering higher-quality optoelectronic devices. The difficulties in high-quality nanostructure synthesis to force a broad library of hybrid nanomaterials with high phase purity and more advanced stability are immensely popular [93]. The importance of synthesis methodologies should be highlighted here; all concerns associated with lateral width, doping concentration, carrier modulation, layer number, and tailoring of electrical and optoelectronic parameters have yet to be addressed, resulting in a decrease in productivity and effectiveness. Obtaining efficient nanomaterials is a crucial matter, requiring scientists to develop novel methods to synthesize them and enable them for excellent performance.

Moreover, nanostructure evolution is still in its early stages. High temperatures, such as more than 700 °C, may, however, cause unwanted processes and defect development at the nanomaterial–nanomaterial interface. In some circumstances, molecular-beam epitaxy may be a better option than CVD. Vacancy generation, interfacial chemical interactions, and layer interaction can all be reduced by using a purer elemental supply, a lower temperature, and an ultra-high vacuum. The ultimate goal is to develop and implement a unified synthesis technique for a wide range of heterostructures that allow for precise performance tuning and control via automated systems, improved production sustainability without employing high temperature and pressure, and cost-effective scalability. Another significant problem in heterostructure synthesis is the layer interactions with substrates and with layers themselves in vdW nanomaterials, which are limited by the intra-layer bonding strength. Dangling bonds are also missing from the surfaces of van der Waals nanocrystals. All of these factors combine to make layered nanomaterial growth, even without the generation of islands, a difficult operation that necessitates careful parameter optimization.

Second, due to its defect source in hybrid nanostructures, the “defects” issue in (nanomaterials)/graphene/Si junctions is also a contentious issue. The significance of defect-free efficient single-crystal production of nanomaterials prior to their assimilation into complicated nanodevice systems should also be underlined. The unintended defects emerge as a result of the fabrication process’s use of defects in metal substrates. A large variety of metal surfaces that can be used in the formation of 2D materials at high alignment facets to develop relatively few flaws during the development of single-crystalline graphene were established in 2020 [94]. This research validates the production of ultrahigh-quality graphene and calls for analogous development of crystal structure nanomaterials on metal substrates, e.g., Al, Ag, ITO, or SiC substrates [95,96].

Following that, impurity doping empowered n-type and p-type semiconductors in Si-technology, marking a watershed moment in the development of high-density integrated circuits. Similarly, alternative ways of doping 2D materials must be investigated for sustainable and industrial-scaled suitable 2D materials. Though scanning techniques, such as TEM, SEM, STM, and spectroscopic techniques, such as FTIR, Raman, and pump-probe approaches for nanomaterial characterization, are ubiquitous, specific scanning or characterization techniques are also required. Scanning atomic electron tomography (SAET) experiments in re-doped 2D TMDs, for example, show three-dimensional (3D) atomic displacements and determine the 3D stain tensor around a particular dopant atom [97]. Because a mismatched atom in the stacked layers is essential for determining the electrical characteristics of 2D heterostructures, it offers valuable insights into forthcoming optoelectronic technology. Furthermore, it clearly shows that phase transformation in 2D materials, including the 1H and 1T phase transformations that were noticed in re-doped MoS_2_, is introduced at only one dopant site, whereas SAET measurement clearly identifies bond distance variations and provides insight into how strain influences the doped edge area [97].

The synthesis compliance of heterostructure optoelectronic devices with the existing CMOS technology is the next issue. A strategy for heterostructure-based devices using the Si CMOS technology was recently developed [98]. Phase transition, top and side contact, and damascene contact are the next orientations for 2D materials, which require more advancements ahead of large-scale optimized contact schemes in order to achieve improved stability and efficiency of these integrated systems. If nanostructures are developed using more advanced nano-fabrication and more modern equipment, nanotechnologies of “more than Moore” implementations of CMOS in sensors, imaging systems, and wireless communications, would be strengthened. The efficient large-scale production of heterostructures is critical for reaching the computing limitations of perceived efficiency. The inability of a scaled transfer technique to construct multiple integrated van der Waals heterostructures of high-quality nanomaterials currently limits this capacity [99]. Although significant advancements in the transfer of wafer-scale have been made, this technology has the potential to cause rip and degeneration in fundamental materials [100]. In addition, a more consistent method for transferring nanostructures on a wider range must be established, or a high-quality and high-performance CMOS process well suited with the growth of heterostructures on undefined substrates must be developed. This should be performed for the unfailing manufacturing of exotic physics deemed by computational development to be optimized developments and/or novel.

Interaction between metal and semiconductor electrodes is also a problem. All optoelectronic devices require a contact surface, wherein a contact with minimized resistance is ideal for large scale application. The traditional metallization process, however, may degrade the intrinsic characteristics of 2D semiconductors due to the fragile heterostructure, resulting in high interface boundaries and excessive contact resistance. Advanced approaches, such as van der Waals metal contact, contact modification, and phase manipulation, have also been suggested and experimentally validated, although sustainability and compliance with industry requirements are still lacking.

The majority of the physical concepts used in nanotechnology come from bulk materials. Richardson’s law, which was derived for bulk materials, is used to describe the thermal radiation method of the carriers over the Schottky barrier in graphene and comparable 2D materials. The classic concept of thermionic emission in 2D heterostructures for the Schottky junction is inadequate because the width of 2D single-layered crystals is equal to or less than the de Broglie wavelength. Additionally, because 2D materials in the transverse position are not very thick, there is no depletion for a 2D p–n junction, specifically for the vertical junctions. As a result, new approaches have been developed to describe carrier transit across these kinds of intersections. Understanding the variations between typical and 2D heterostructure-based optoelectronics, as well as the function of hot-electron and excitonic phenomena in the heterostructure photoresponse, is critical. The fundamental responsiveness of various types of heterostructures is influenced by their structural system, as well as the hot electron relaxation process, for example, if the dominant hot-electron cooling process at the junction is identical, the graphene-metal junctions can also display the equivalent photoresponse phenomenon [101,102,103,104,105]. Two identical 2D connections may react differently depending on their quality, interface, and fabrication approach. Thus, instead of focusing just on the structural system and band structure orientation, it is critical to classify 2D optoelectronic devices based on their intrinsic responsiveness and prevailing electron cooling mechanism. To enhance optical sensing performance in 2D heterostructures, an arrangement based on a variety of hot carrier mechanisms was developed, followed by the development of a device fabrication fit for the dominant mechanism, wherein PTE and bolometric effects are mostly used for the in-plane geometrical extraction of hot electrons [101,102,103,104,106]. Whereas, the PTI effect is used to extract hot carriers vertically [107,108]. However, various methods can possibly coexist in a single device. As a result, developing experimental methodologies and strategies to differentiate the impacts of various hot electron-based processes in 2D heterostructures is very important in future research. Another important subject to investigate in this regard is whether the electron extraction phenomenon via the PTI method is more efficient in 2D-2D or 2D–3D heterostructures. Exciton generation and separation are critical phases in determining photocurrent response in TMD-based 2D heterostructures. Despite several investigations into exciton behavior in 2D heterostructures and the van der Waals stack, studies on the mechanics of exciton production and separation remain limited due to crystalline structure, layering pattern, and layer count. A clear understanding of spectroscopic and photocurrent analysis is important to be considered for the experimental research of photocarriers in TMD based-heterostructures. The dynamics of inter-layer energy and charge transport in 2D heterostructures and homo-structures are understudied, as well. From a more fundamental standpoint, it is critical to comprehend the significance of electron momentum disparity between successive layer dependent on layering orientation, as well as how it reflects both the phenomenal effects of PTE and PTI. The charge and energy interaction among both uncorrelated and correlated layered heterostructures has recently received a lot of interest [78,109,110,111], leaving the door open for more research, such as how thermal energy is distributed in a system when the layer interface fluctuates from strong to weak.

The harvesting of nanomaterial parameters for optoelectronic applications is another difficulty. For optical sensing systems, this relates to when it is necessary to attain both high speed and high sensitivity IR optical sensing, which are typically not attainable in one device. Dynamic nature, strong interactions between light and matter, and a gate-tuneable Fermi level are all advantages of the nanoscale 2D materials, which can be utilized as optical sensing channels, which could lead to hypersensitive and high-speed optical sensing systems. However, the heterostructure-based optical sensing systems’ high performance and detectivity can be achieved with an extremely slow response time, wherein the insulating barrier inserted heterostructure is used to improve sensitivity by suppressing the large amount of dark current. Optical responsivity is one of the most important elements to consider when evaluating optical sensing systems’ efficiency, which ranges from UV-Vis to FIR. Considering long wavelength detection necessitates narrow bandgap semiconductors to capture light, and recent achievements have been made in the exploration of novel outstanding 2D heterostructures. Besides the unearthing of graphene, the growth of 2D heterostructure technology has been accomplished, resulting in the foundation of photoactive materials used for optical sensing systems, evolving from graphene to black phosphorus, narrow bandgap TMDs, and other photoactive 2D materials. Research has primarily concentrated on enhancing the optical responsivity and throughput of optical sensing systems for device performance. In this regard, various performance metrics, including photoresponsivity and gain, are enhanced dramatically as a result of concerted initiatives [1,2,3,91,92]. Other key parameters that are critical to actual PD applications, such as NEP, detectivity, and broad detection wavelength range, require more effort.

Integrating hybrid materials with 3D structures is a feasible option for commercializing 2D materials at the moment because each part has unique functionality. Integrating 2D materials with fabricated Si ICs, particularly in PDs, results in different activities, with light interaction occurring in 2D materials and data processing occurring in Si-based ICs. Because these devices, which include optical sensing systems, sensors, and filters, have comparable performance to existing conventional devices, incorporation of such devices with suitable predictable semiconducting materials is required to fully understand the mechanism and working potential of 2D–3D hybrid devices.

Neuromorphic computing technology is a promising optoelectronic discipline in which large neural networks implement neurological functions, such as object and speech recognition and self-learning [112]. Optical sensing systems have immense potential as optical synaptic sensors in optical neural networking, imitating retinal processes and reducing power usage for such efficient neuromorphic processing. As a result, it is critical to investigate the efficiency of 2D photosensors in immense neural network models and find image processing measures to tackle device variability caused by nanomaterial defects.

Modern optoelectronics could benefit from hybrid nanostructures based on hybrid 2D QD-based heterostructures [113]. Nonetheless, obstacles remain. For example, owing to the low optical absorption and quick photogenerated recombination of electron–hole pairs, the optical detectivity of 2D materials is constrained. One strategy for improving light absorption is surface engineering through surface defects or bandgap adjustment. However, this is usually a difficult technique. Since 2D materials are found to be compatible with QDs, the hybrid 2D QD-based heterostructures may provide an alternative approach to enhance device performance as compared to other existing semiconductors. Furthermore, because of their adaptability and flexibility, these hybrid heterostructures could aid in the successful isolation of electron–hole pairs.

The technical constraints of developing hybrid nanostructures for nanoscale materials now necessitate increasingly advanced methods. Concepts on high-performance advanced future optoelectronics, such as artificial intelligence [114], machine learning [71,78,115,116], or photovoltaic and energy harvesting [117], are being developed as a first-step toward the bright future of 2D heterostructures [71,78,114,117]. In this case, the influence of hybrid nanostructures for the efficient integration of such materials is essential, resulting in the advancement of physicochemical interactions among various compounds in an integrated system and the emergence of revolutions in medical and industrial technology, as well as artificial intelligence training and other technological innovations.

Generally, hybrid structures are critical for the advancement of optoelectronic functionalities, such as valleytronics [118,119,120], twistronics [101,102,121,122,123,124,125,126,127,128], and spintronics [129,130], as well as neuromorphic devices [71,131,132,133,134,135,136,137,138]. The strength and stability of the bonding at hybrid nanostructure surfaces [99] are some of the problems of hybrid nanostructures, and they warrant more investigation. Addressing interlayer interactions could also lead to new ways to design the 2D layered systems of optical sensing, superconductivity, and spin filtering effects. The aim of hybrid nanostructure research is to design large-scale production and incorporate processes that approach the efficiency of existing Si technology. Even though there have been several major breakthroughs in many fields of optoelectronics that are constrained to single devices and narrow networks based on nanostructures, evident and insurmountable barriers remain, including material synthesis, device fabrication, and its assimilation. To produce high efficiency 2D integrated circuits, several shortcomings must be addressed, including low temperature van der Waals heterostructure synthesis strategies, an efficient charge transfer mechanism, persistent dielectric 2D material layer deposition, and consistent metal connections. Separate synthesis processes for 2D materials are currently not scientifically or economically feasible. As a result, the optimal scenario for commercializing the van der Waals heterostructure device is for these materials to be produced on existing Si fabrication processes. Consequently, we expect more research to focus on developing methodologies and device strategies for integrating 2D materials with conventional Si technology.

**Figure 13:**
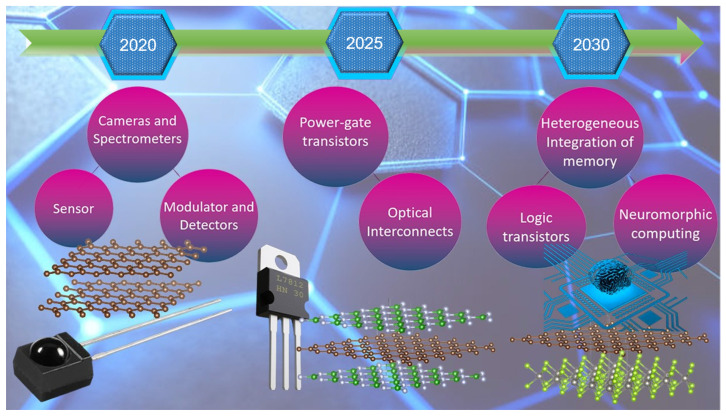
The prediction of the development roadmap of future modern optoelectronic systems.

Last, but not least, some representative optoelectronic systems in modern optical sensing are highlighted and predicted for future trends until 2030, such as the power–gate transistors, optical interconnects, logic transistors, heterogeneous integration of memory, or neuromorphic computing (Figure 13). These could potentially emerge as the foremost developments regarding attention in the future with regard to the research community.

## Data Availability

Not applicable.

## Abbreviations

1D	One-dimension
2D	Two-dimension
3D	Three-dimension
Si	Silicon
UV	Ultraviolet
IR	Infrared
NIR	Near infrared
CMOS	Complementary metal-oxide-semiconductor
TMDs	Transition metal dichalcogenides
NEP	Noise equivalent power
Au NPs	Gold nanoparticles
rGO	Reduced graphene oxide
PhC	Photonic crystal
Quality factor	Q-factor
TOS	Tungsten oxyselenide
ns	Nanosecond
ps	Picosecond
fJ	Femto-joule
BP	Black phosphorous
R (λ, D)	Photo-responsivity (the incident light wavelength and the external biasing displacement field)
ORRAM	Optoelectronic resistive random-access memory
ANN	Artificial neural network
CVD	Chemical vapor deposition
FEO	Functional electrical optics
TENG	Triboelectric nanogenerator
vdW	van der Waals
TEM	Transmission electron microscopy
SEM	Scanning electron microscope
STM	Scanning tunneling microscope
FTIR	Fourier-transform infrared spectroscopy
SAET	Scanning atomic electron tomography
PTE	Photo-thermal emission
PTI	Photo-thermionic
Si-based ICs	Si-based integrated circuits
PDs	Photodetectors
QDs	Quantum dots
